# Supplementary material for: Evaluation of the accuracy of the IDvet serological test for Mycoplasma bovis infection in cattle using latent class analysis of paired serum ELISA and quantitative real-time PCR on tonsillar swabs sampled at slaughter
Source: PLoS One. 2023 May 11;18(5):e0285598. doi: 10.1371/journal.pone.0285598 (PMC10174590; doi:10.1371/journal.pone.0285598)
Supplement: S1 Table — (PDF) [file pone.0285598.s001.pdf]

ELISA Threshold	Island	PCR result	ELISA pos	ELISA neg	Total
60	North	PCR pos	137	55	192
		PCR neg	1051	17672	18723
		PCR comment	11	23	34
		total	1199	17750	18949
	South	PCR pos	1145	235	1380
		PCR neg	3219	34873	38092
		PCR comment	27	55	82
		total	4391	35163	39554
70	North	PCR pos	129	63	192
		PCR neg	859	17864	18723
		PCR comment	8	26	34
		total	996	17953	18949
	South	PCR pos	1093	287	1380
		PCR neg	2797	35295	38092
		PCR comment	24	58	82
		total	3914	35640	39554
80	North	PCR pos	122	70	192
		PCR neg	709	18014	18723
		PCR comment	8	26	34
		total	839	18110	18949
	South	PCR pos	1015	365	1380
		PCR neg	2349	35743	38092
		PCR comment	21	61	82
		total	3385	36169	39554
90	North	PCR pos	108	84	192
		PCR neg	557	18166	18723
		PCR comment	6	28	34
		total	671	18278	18949
	South	PCR pos	944	436	1380
		PCR neg	1968	36124	38092
		PCR comment	18	64	82
		total	2930	36624	39554
100	North	PCR pos	100	92	192
		PCR neg	421	18302	18723
		PCR comment	6	28	34
		total	527	18422	18949
	South	PCR pos	843	537	1380
		PCR neg	1619	36473	38092
		PCR comment	16	66	82
		total	2478	37076	39554
